# A Lesson from New Zealand

**Published:** 1908-12-05

**Authors:** 


### A Lesson from New Zealand.

"The wise man," remarks Father Fuller in one of his classical sermons, "learns equally by the mischances of others and the fortune that falls to his neighbours. . . . For there is a lesson in avoidance as there is one in accomplishing." And if he wanted an example in point the Elizabethan master of prose writing, had he lived in our day and chanced upon the latest annual report of Dr. Mason, the Medical Officer of Health for the Dominion of New Zealand, could scarcely have found a better than the state of vaccination in that part of the Empire. To the profession in England Dr. Mason's report cannot fail to be interesting, for it shows, more forcibly perhaps than any other official document which has hitherto been published, what might be the results were the conscientious clause made so elastic as to render the vaccination laws a dead letter. The tendency towards allowing the individual greater liberty of action in matters which affect the health of the community is a growing one, even amongst the section that flaunts its alleged Socialism as a guiding principle in its politics. The recent alterations in the vaccination laws have not been progressive in any true sense, as we pointed out at the time when they were brought forward. At the same time the condition of affairs here is not so discouraging as it appears to be in New Zealand. That country, to judge from Dr. Mason's report, must be veritable Islands of the Blest for the anti-vaccinator. There, as Dr. Mason justly remarks, the matter is entirely in the hands of the parent. "He may know nothing about the question—that matters not. He is not required to show even an intelligent appreciation of the pros and cons of the position. He has simply to go before a magistrate and say 'I believe it will injure my child,' and the child goes unprotected. Last year out of 24,321 children born only 4,486 were vaccinated. Over 81 per cent. of children born in 1906-7 are unprotected against small-pox. But that is not the worst of the question. Apparently a large section of the community are passively breaking the law while a noisy few are actively opposing it. . . ."

This is, in the words of the High Commissioner, a pretty state of things, and the most melancholy part of it is that it is the ideal which a large section in this country appears to be striving for. Anti-vaccination is making headway, strange as it may seem to those who are firmly convinced of the hopelessness of struggling against facts. The present generation of parents, as Dr. Mason observes, little knows what a fateful disease small-pox is. Ninety-nine per cent. of our population have never seen a case or the results of the disease. They do not consider, therefore, that the passing inconvenience that attends vaccination is a sufficiently small price to pay for a comparative immunity against one of the most loathsome and horrible of the diseases that afflict mankind. Few of us—and the "man in the street" least of all—emulates the philosophy of Ruskin, and wonders not at what men suffer but at what they escape. The result is that the community has its health imperilled by a section of faddists and sentimentalists, and that the anti-vaccinationist looms large in the political horizon and "worries excessively until he succeeds." Those of us who have any knowledge of small-pox and its history, those, too, who have read Dr. Mason's report and marvelled at the inaction of a colonial government that calmly submits to the humiliation of having its laws transgressed and unobserved, will not contemplate that success with equanimity. And yet, unless every medical man makes it his personal duty to teach his patients the truth about vaccination, none of us can be astonished if the conscientious objector wins all that he has fought for—the right to become a danger to his fellow men, the privilege of posing as a martyr while he is not far short of a murderer, and exemption from a measure that has been made compulsory on the rest of the community.

